# Inferring Epidemic Network Topology from Surveillance Data

**DOI:** 10.1371/journal.pone.0100661

**Published:** 2014-06-30

**Authors:** Xiang Wan, Jiming Liu, William K. Cheung, Tiejun Tong

**Affiliations:** 1 Department of Computer Science and Institute of Theoretical and Computational Study, Hong Kong Baptist University, Kowloon Tong, Hong Kong; 2 Department of Computer Science, Hong Kong Baptist University, Kowloon Tong, Hong Kong; 3 Department of Mathematics and Institute of Theoretical and Computational Study, Hong Kong Baptist University, Kowloon Tong, Hong Kong; Northeastern University, United States of America

## Abstract

The transmission of infectious diseases can be affected by many or even hidden factors, making it difficult to accurately predict when and where outbreaks may emerge. One approach at the moment is to develop and deploy surveillance systems in an effort to detect outbreaks as timely as possible. This enables policy makers to modify and implement strategies for the control of the transmission. The accumulated surveillance data including temporal, spatial, clinical, and demographic information, can provide valuable information with which to infer the underlying epidemic networks. Such networks can be quite informative and insightful as they characterize how infectious diseases transmit from one location to another. The aim of this work is to develop a computational model that allows inferences to be made regarding epidemic network topology in heterogeneous populations. We apply our model on the surveillance data from the 2009 H1N1 pandemic in Hong Kong. The inferred epidemic network displays significant effect on the propagation of infectious diseases.

## Introduction

Recent outbreaks of infectious diseases have stressed the urgency of effective research on the dynamics of infectious disease spread over geographical regions and in various populations [1]. The pandemic of influenza A (H1N1) in 2009 struck more than 208 countries and territories experienced the pandemic, collectively causing at least 12,799 deaths [2]. Great benefits would be gained from the rapid formulation of appropriate control policies to contain the spread of the infectious disease and eliminate it from the population. However, the complex dynamics of infectious disease spread poses a significant challenge to the design of a realist control strategy. Computational modeling has long been an important tool for understanding spread patterns of infectious diseases, predicting outbreak severity, evaluating the efficacy of interventions, and optimizing the deployment of new control policies. The majority of disease models are based on a compartmental model called the Susceptible-Infected-Recovered (SIR) model [3]–[6]. It studies the spread of infectious diseases by tracking the number (S) of people susceptible to the disease, the number (I) of people infected with the disease, and the number (R) of people who have had the disease and are now recovered. Assuming the population mixes at random, three ordinary differential equations are defined for 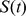, 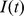, and 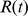 at time 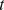: 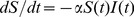
(1)




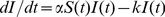
(2)




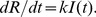
(3)


Here, 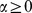 is the effective transmission rate and 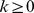 is the recovery rate. The value of 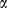 is a key indicator for the guidance of implementing control and intervention policies.

The SIR model and its variants are appropriate for modeling the temporal dynamics of epidemics in the randomly mixed population [7]–[9]. However, it is difficult to use such models to investigate complex social structures or mixing patterns that depend on network structure. Network epidemic models represent an alternative to compartmental models that can more easily capture the effects of social structure. An epidemic network consists of a set of nodes and a set of links that connect them, where the nodes correspond to spatial locations with reported (or observed) disease incidences over time and the directional links indicate the probability (or likelihood) of disease transmission from one node to another over time. It can be used to characterize the temporal-spatial patterns of disease transmission. Determining an accurate epidemic network requires knowledge of every individual (or host) and every relationship between individuals. A detailed review [10] summarizes four major types of models, including patch models [11]–[13], distance-transmission models [14], multi-group models [15], [16], and network models [17]. However, for all but the smallest population, collecting individual-level data is an impractically time-consuming task. To bypass the difficulties of collecting data, researchers started to investigate several types of computer-generated networks in the context of disease transmission in population-scale studies [18]–[22]. Given the mean-field theory, they have proposed to model epidemic spread in scale-free networks. However, it remains an open question whether real networks are close to scale-free, or only scale-free over a finite domain [23]. The dynamics of infectious disease spread rely strongly on the structure of the epidemic network topology.

Related topics, such as social influence through networks, the diffusion of innovations, and information propagation, have also been studied in the context of various disciplines including economics [24], public health [25], scientific publishing [26], and virus propagation [27]. However, each of these models has one or more aspects that are problematic in studying the temporal-spatial dynamics of infectious disease spread. Some do not capture the probabilistic nature of infection while others make assumptions about the types of interactions occurring between individuals that are often not valid in the context of disease transmission. How to infer the epidemic network topology remains a challenging research topic.

To accurately catch when and where outbreaks emerge at the first time, one approach at the moment is to implement surveillance systems in regional or national health and medical centers. The accumulated surveillance data including temporal, spatial, clinical, and demographic information, can provide valuable information with which to infer the underlying epidemic network of infectious disease spread. In this work, we introduce a new computational model that can discover the epidemic network of infectious disease spread from the surveillance data. In our proposed model, the dynamics modelled in the classical SIR model is described by an inhomogeneous Poisson process characterized by a piecewise rate function, and the spatial relationships are characterized by interactions of multiple inhomogeneous Poisson processes in a network. Our proposed model allows inferences to be made regarding the progression patterns of infectious diseases in heterogeneous populations. We apply our model on the surveillance data from the 2009 H1N1 pandemic in Hong Kong. The inferred epidemic network displays significant effect on the propagation of infectious diseases, and is useful to public health authorities in predicting the influence of future prevalence and the implications of control polices.

## Materials and Methods

Classic modeling of infectious diseases assumes that the population is well-mixed. However, this assumption is unrealistic for many diseases with spatial spread patterns. Here we first describe the dynamics of classical SIR models through an inhomogeneous Poisson process and then formulate a new stochastic network model that explicitly considers the geographical structure to capture the temporal-spatial dynamics of infectious disease spread in heterogeneous populations.

### Poisson process for modeling the dynamics of classical SIR models

There is no analytic solution to solve SIR-type dynamics without making approximations. To model the dynamics in continuous time, the discrete-time models are often used with a given time interval 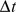. Let 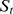, 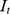, and 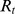 be discrete random variables for the number of susceptible, infected, and recovered individuals at time 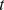. Using the Euler method, the SIR model for the sub-population 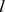 can be rewritten as three equations: 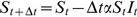
(4)




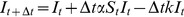
(5)




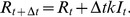
(6)


In [28], [29], the progression of disease spread is characterized by tracking the number of 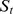 with a chain binomial model. The number of susceptible members 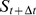 (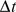 represents the infectious period of the disease and is always chosen to be 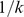) at time 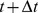 is a binomial random variable that depends on 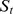 and 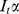, 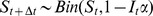, which provides a recursive relationship between 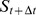 and 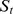 and produces a formal stochastic process. We use an alternative approach to model the dynamics of infectious disease spread. In an epidemic outbreak, the number of new infections during a time interval is of major concern. Let 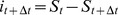 be the number of new infections between time 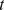 and time 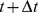. Let 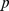 be the probability that a contact between a susceptible and an infected individual results in a new infection and 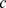 is the average number of susceptible members to whom an infected individual may spread the disease at time 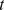. The infectious individuals 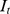 are assumed to infect susceptible members 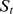 only at time 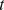. After that time, they are no longer infectious. This is reasonable because patients, once confirmed as infected, will have much less possibilities to spread the disease since they may start the treatment, take rest at home, adopt some measure to prevent the disease spread (such as wearing a face mask outside), or be quarantined. With this assumption, we have the following proposition.

### Proposition 1



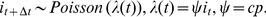
(7)
**Proof**: Given one infected person and one person in the population, the probability they meet each other is 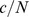. Then the probability that the contact results in an infection is 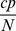. Given the infected person, the number of new infected people in 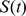 is 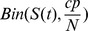. Since 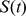 is large, 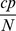 is very small, and 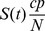 is finite, we can approximate 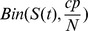 with 
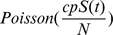
. Because 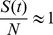, we get that given one infected person, the number of new infected people follows 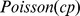. Then given 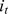 infections at time 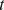, the number of new infections 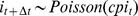 (assume the social contacts of infected people are independent of each other).

The above equation (Eq.(7)) defines an inhomogeneous Poisson process - a stochastic counting process characterized by an intensity function [30]. It has an advantage over the chain binomial models in the dynamic modeling of infectious disease spread. In the chain binomial models, varying the selection of 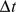 can distort the dynamic patterns [31]. In contrast, the Poisson distribution can be freely adjusted with respect to 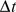 without affecting the stochastic process due to the Poisson property. Motivated by this advantage, we propose a new stochastic network model of infectious disease spread.

### Network modeling of infectious disease spread

Consider the surveillance data 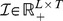 from 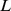 locations. Each element 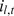 of 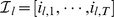 corresponds to the number of new infections between time 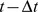 and time 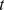 at location 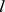. Let 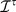 denote the transpose of 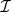 and then 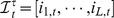 correspond to the new infections at all locations between time 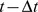 and time 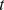. Ignoring the effects of network structure, the dynamics of 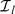 can be modeled as the inhomogeneous Poisson process defined in Eq.(7). In the network modeling, we need to capture both the dynamics of the Poisson process at every location and the dependency among multiple Poisson processes at different locations linked in a geographic network.

**Figure 1:**
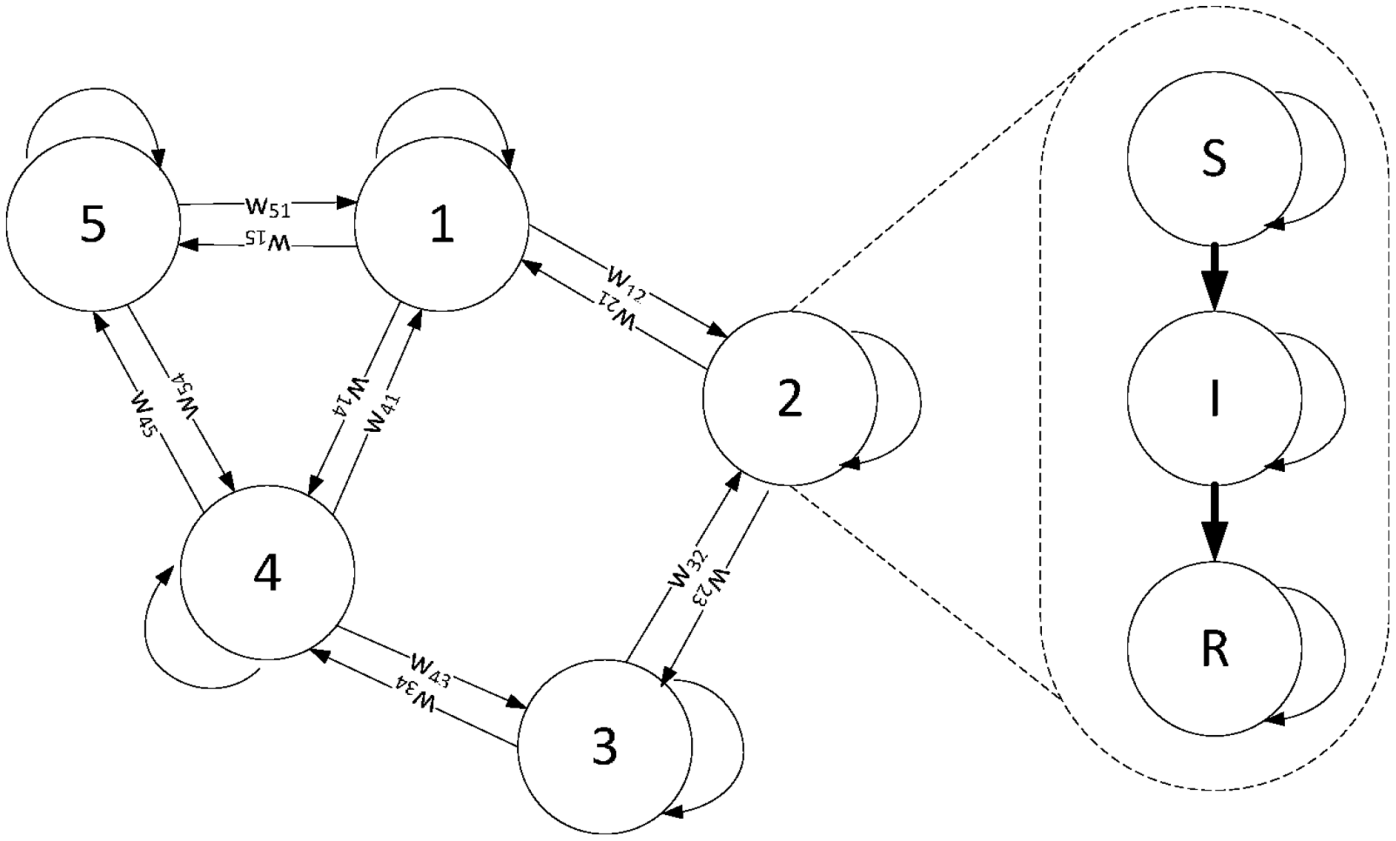
A toy example of infectious disease spread in a five node network. Each node represents a physical location associated with an inhomogeneous Poisson process shown as an example for node 2. Each edge is associated with 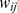 measuring the spreading trend from node 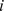 to node 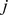.

We use a directed graph 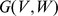 to represent a geographic network, where 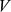 is the set of nodes and 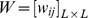 (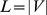) is the adjacency matrix of the graph. Each node indicates a sub-population in one location. Each 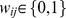 indicates the existence of infection spread from node 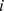 to node 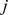. Figure 1 provides a toy example of a five node network. Each node 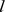 is associated with an inhomogeneous Poisson process characterized by an intensity function 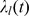 and its own 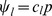 (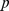 is a constant for a specific infectious disease). Let 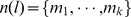, where 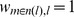, represent the adjacent nodes of node 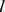 in the disease spread network. The spatial interactions will change the intensity function of each node. Thus 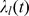 will not only depend on 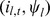 but also be associated with 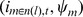.

We consider a generalized linear model (GLM) for the intensity function 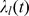 with respect to 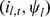 and 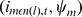. The rate of the Poisson process defined in Eq.(7) is rewritten as 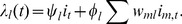
(8)


The values of 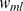 give rise to a transmission network of infectious disease across different locations. For a specific location 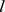, the value of 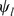 measures the speed of disease spread caused by internal infections and the value of 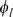 measures the speed of disease spread caused by external infections (or imported infections). Our goal is to estimate 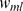, 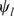, and 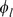 using the surveillance data 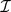.

### Parameter estimation

**Figure 2:**
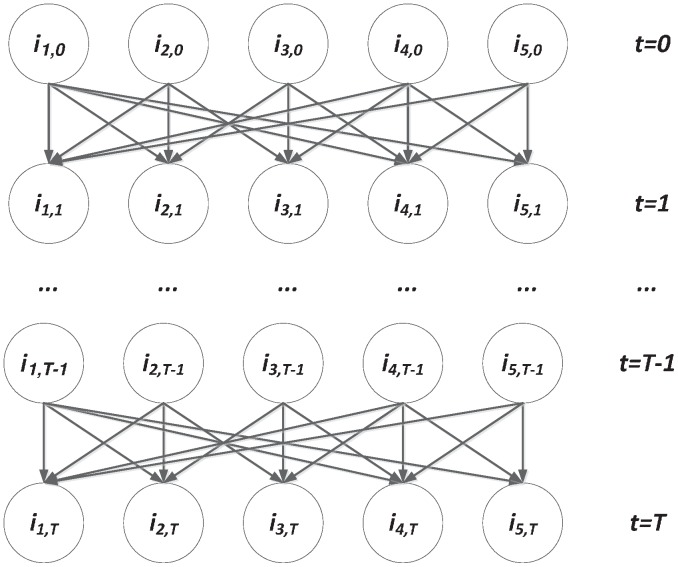
The Markov network, reduced from the spreading network in Figure 1 with the independence assumption. The state of each node 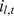 is the number of new infections at location 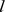 (node 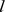 in Figure 1) at time 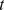. The states of nodes at time 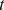 are independent and only dependent on the states of nodes at time 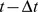.



In principle, it is intractable to infer the parameters in Eq.(8) on an arbitrary network because the conditional distribution 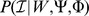 is computationally too expensive to obtain. To make the inference tractable, the 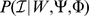 is factorized with an independence assumption, which is that the states of nodes at time 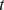 are independent and only dependent on the states of nodes at time 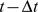. This assumption has been widely applied in the area of machine learning to factorize an exact joint probability distribution into a multiplication of many marginal probability distributions [32]. Then the dependency graph shown in Figure 1 is reduced into the Markov network shown in Figure 2. Consequently, the 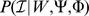 is defined as

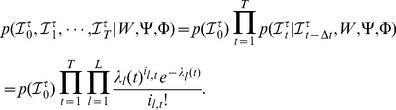
(9)Our target is to find the following maximum likelihood estimators: 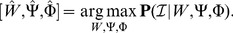
(10)


It can be easily proven that the negative 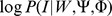 is a biconvex function of 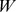, 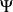, and 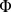, which means that the negative 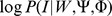 is a convex function of 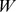 if 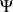 and 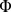 is fixed, and vice versa. Given 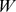, it is straightforward to estimate 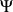 and 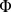. However, it is still challenging to infer 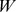 for the given 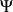 and 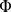. Although there are many popular optimization approaches for bi-convex problems, they can not be applied in our work because our model is non-continuous and non-differential while most available approaches are gradient based methods. Here we use the genetic algorithm to solve this issue.

Genetic Algorithms (GAs) are adaptive heuristic search algorithm based on the evolutionary ideas of natural selection. They have many advantages to solve problems where candidate solutions can be described with the chromosome encoding. Finding the network topology is one such problem where one network topology can be viewed as the chromosome of one individual in a generation. The basic concept of GAs is to simulate processes of survival of the fittest. They represent an intelligent exploitation of a random search within a defined search space to solve a problem. Experiments show that many such problems, which prove difficult for traditional methods, are ideal for GAs [33]. We design a genetic algorithm (please see Table 1) to infer the network structure 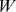. The only input to the GA algorithm is the surveillance data (the number of new incidences in a sub-population during a time interval). The GA algorithm starts from the first generation - a pool of randomly generated adjacency matrix. Based on the evolutionary theory, individuals in subsequent generations can be generated using the typical GA operators (crossover and mutation) and be selected in a way that resembles the natural selection. The crossover (also called recombination) operator is to produce a child individual of the next generation from two parent individuals of the current generation. The mutation operator is to randomly change some parts of the new generated individual. Please check [33] for more details about genetic algorithms. The output is the adjacency matrix with the best value of the fitness function in the last generation.

**Table 1:** Genetic Algorithm.

1. Randomly generate an initial population M(0) of the network structure and for each 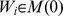 , estimate 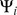 and 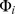 using Eq.(9) and Eq.(10) and use the computed maximum likelihood as the fitness of 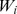 .
2. Copy the top 10 percent of 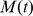 into 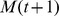 .
3. Randomly choose four network structures from 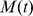 as parents and use crossover operator to generate two child network structures.
4. Conduct the mutation for both generated child network structures.
5. Compute the fitness of two generated child network structures and save the better one in 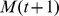 .
6. Repeat Step 3–5 until the capacity of 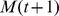 is full.
7. 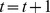 .
8. Repeat Step 2 until the new generated population does not improve the fitness value.
9. Output the adjacency matrix with the best value of the fitness function in the last generation.

## Results and Discussion

### Case study

**Figure 3:**
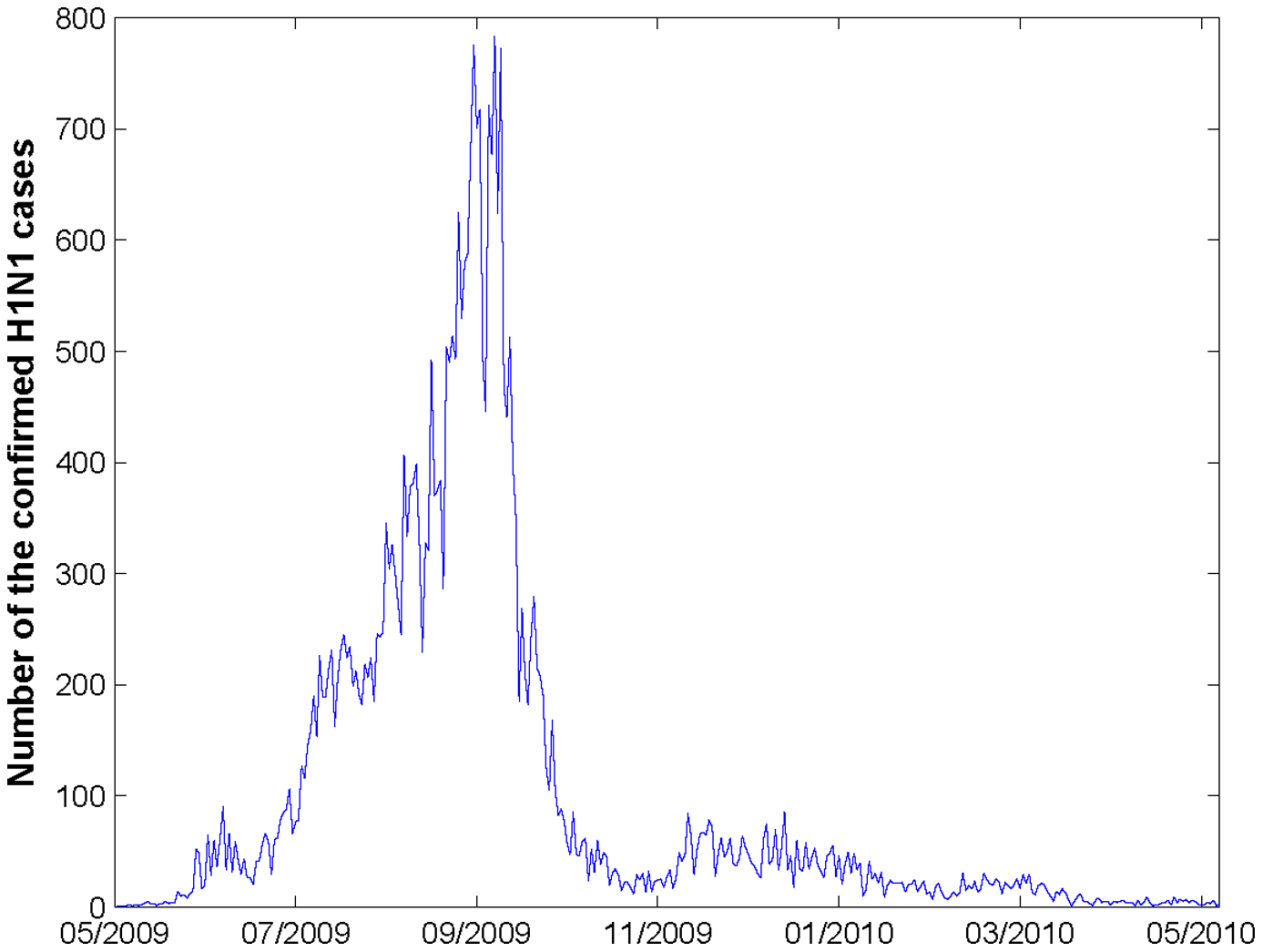
Daily H1N1 epidemic curve in Hong Kong from May 1, 2009 to May 23, 2010. The epidemic curve of confirmed H1N1 cases reaches its peak at the end of September, 2010.

In the case study, we apply our model on the surveillance data from the 2009 H1N1 pandemic in Hong Kong. We have acquired the time series data of daily number of confirmed H1N1 cases with symptom onset from May 1, 2009 to May 23, 2010. The database includes 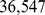 confirmed cases with demographic information on location, age, and sex along with the laboratory-confirmation dates. The epidemic curve of confirmed H1N1 cases (see Figure 3) reaches its peak at the end of September, 2009, after which the intervention procedure comes into effect and the curve goes down. We use the data up to Sept 30, 2009 including 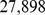 cases (more than 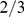 of all cases).The infectious period 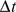 of H1N1 is set 3 days.

**Figure 4:**
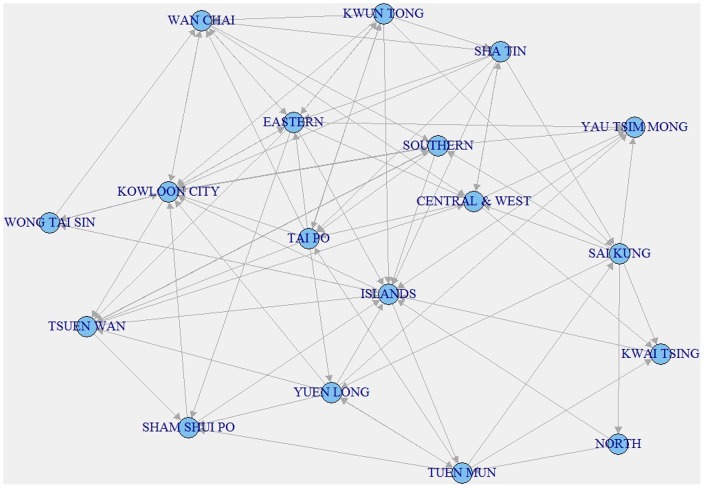
Computed spreading network of 2009 H1N1 in Hong Kong.

Hong Kong is geographically divided by 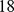 political areas (districts). Each district is considered as one node in the epidemic network. The learned epidemic network in Figure 4 show how the H1N1 spreads in the geographical network of Hong Kong. To examine the effect of epidemic network topology in the spread of H1N1, we compare the following models:

- M1: 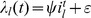. M1 is an independent homogeneous model where the infectious disease spreads independently and at the same internal growth rate in different locations.
- M2: 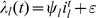. M2 is an independent heterogeneous model where the infectious disease spreads independently but at the different internal growth rates in different locations.
- M3: 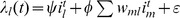. M3 is a dependent homogeneous model with uniform network effect where the infectious disease spreads dependently with the same external effect and at the same internal growth rate in different locations.
- M4: 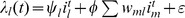. M4 is a dependent heterogeneous model with uniform network effect where the infectious disease spreads dependently with the same external effect but at the different internal growth rates in different locations.
- M5: 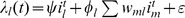. M5 is a dependent homogeneous model with non-uniform network effect where the infectious disease spreads dependently with the different external effects but at the same internal growth rate in different locations.
- M6: 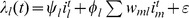. M6 is a dependent heterogeneous model with non-uniform network effect where the infectious disease spreads dependently with the different external effects and at the different internal growth rates in different locations.

Table 2 summarizes the results for different model formulations. In this paper, the model selection is conducted mainly based on the likelihood ratio test, which is often used to compare the fits of two nested models, one of which (a reduced model) is a special case of the other (the full model). The likelihood ratio of two models can be used to compute a p-value which is then compared to a critical value to decide whether to reject the reduced model in favour of the full model. However, for two models which are not nested (for instances, M4 and M5), we have to use other assessments. AIC [34] and BIC [35] are two popular choices. AIC denotes Akaike Information Criterion that deals with the trade-off between the goodness of fit of the model and the complexity of the model. BIC denotes Bayesian Information Criterion (BIC) that is closely related to the AIC but penalizes the complexity of the model (the number of free parameters in the model) more strongly. Both assessments in the model selection have advantages and disadvantages. Therefore, we report both AIC and BIC in Table 2. In most of the times, two assessments agree on the preferred model.

**Table 2:** Results of the analysis of 2009 H1N1 epidemic in Hong Kong.

	AIC	BIC	Log 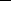 likelihood	Compared model	Benefit (P 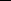 value)
M1	5191.36	5205.39	−2592.58		
M2	5222.34	5315.83	−2591.17	M1	1.00
M3	5177.47	5196.17	−2584.73	M1	0.005
M4	5150.36	5284.53	−2554.18	M2	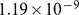
**M5** ^*^	4821.7	4916.86	−2389.85	M3	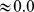
M6	4841.49	5019.12	−2382.74	M4	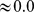

The P 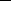 value is computed by performing the likelihood ratio test between two models in comparison. M5 is the best model that fits the data.

**Figure 5:**
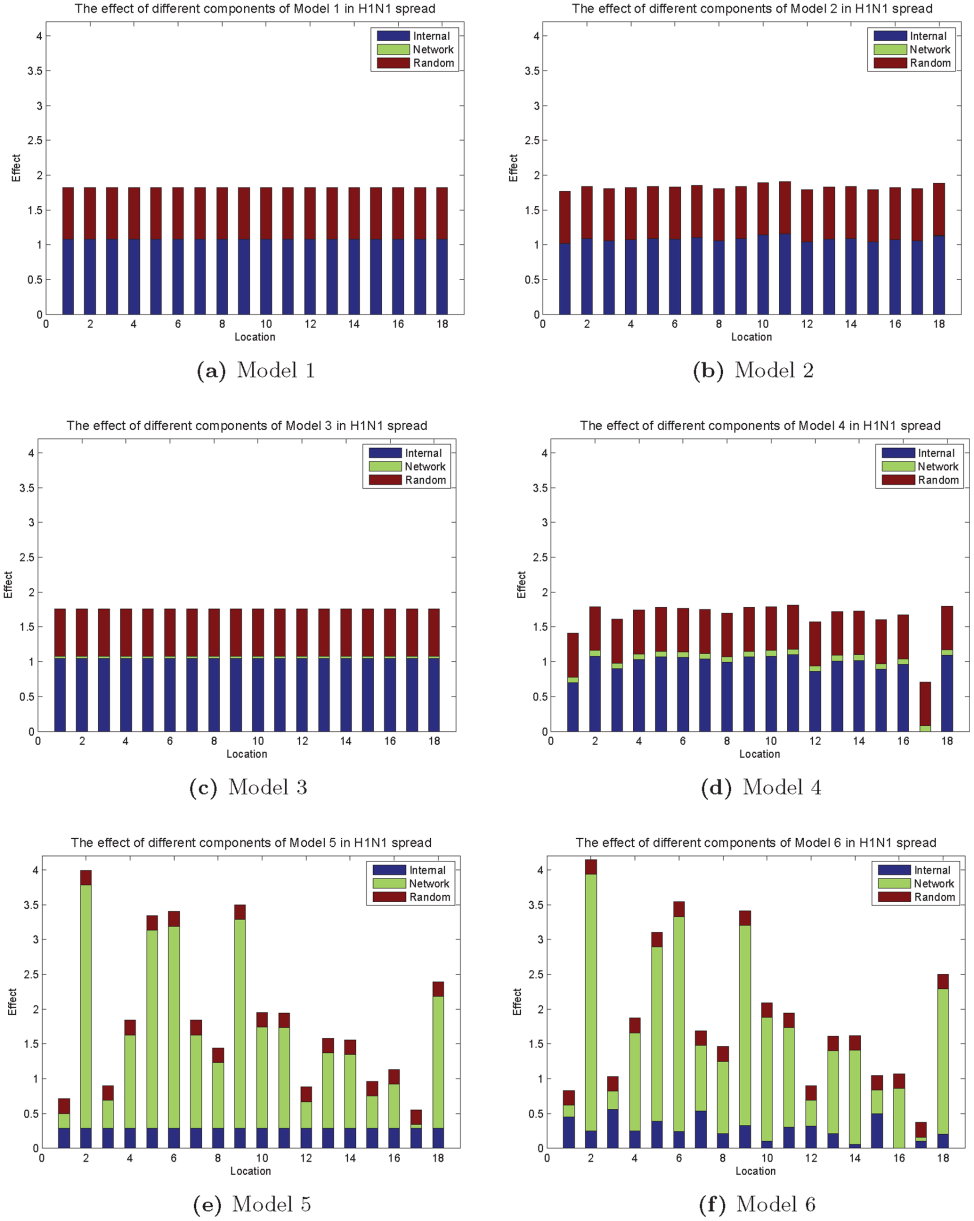
The comparison of different effects in 2009 H1N1 spread in Hong Kong. The internal effect is the exponential value of 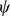 or 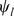. The network effect is the exponential value of 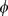 or 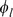. The random effect is the exponential value of 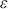.

The comparison between M2 and M1 indicates that if the network effect is ignored, the disease spreads at the same growth rates for different locations in Hong Kong. Hong Kong is one of the most densely populated places in the world. Although population varies in different districts, the concentration of people is high in all districts due to the fact that more than 75 percent of Hong Kong area comprises no-built-up areas. Therefore, if each district is examined individually, the density of population in the living space will be the main factor to affect the spread rate of infectious disease. However, once the network topology is taken into consideration in disease spread, different locations show different spreading patterns. Both M4 and M3 provide a better fitness over M2 and M1. The significant benefit of M5 over M3 and M6 over M4 indicates that the network effect, which measures the imported infection, varies between locations. This is mainly due to people's daily travels. Hong Kong possesses a heavy heterogeneous traffic pattern. Therefore, the imported infections vary significantly among different locations. Figure 5 illustrates the effect of different components of all models. We can see that if the network effect is considered for each location, the models M5 and M6 can explain the data very well. The random effect only accounts for a small portion in the explanation of disease propagation within the different locations, which indicates that our approach is an empirically feasible solution in the analysis of future epidemic in Hong Kong. There is no benefit of choosing M6 over M5 (P 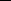 value = 0.982). We can see in Figure 5 that in comparison with the network effects, the internal effects play a very small role in explaining the data in both M5 and M6. In Hong Kong, there are intensive transits between districts and as a result, the network effects dominate the epidemic and already explains most variations in the disease spread. Therefore, there is little benefit gained by looking detailed into the internal differences.

How to verify the inferred network topology remains an unresolved issue because the true epidemic network topology is unknown. To our knowledge, the best way to do so is to use the contact data among some infected patients to verify the results. However, such data is not always available and sometimes difficult to collect due to many issues (such as privacy). In our work, we infer the epidemic network topology based on model selection and make the decision from the statistical point of view. Researchers have shown that the spatial spread of infectious diseases has a high correlation with the human mobility both on a large and short scale [36]–[39]. The inferred epidemic network topology in our work displays such correlations. Some locations that have high connectivity in Figure 4, such as Kowloon City, Central&West, and Eastern, are transit centers in the public transportation network of Hong Kong.

## Conclusion

In this paper, we have developed and demonstrated a computational model that extracts the epidemic network topology from the surveillance data of infectious diseases. Especially for disease spread in non-random mixing populations, heterogeneity is very likely exist and should be accounted for. This is done by including region-specific spreading patterns in a stochastic network model. The proposed model distinguishes itself from previous studies in fundamental ways:

- The dynamics of the classical infectious disease model are described by an inhomogeneous Poisson process characterized by a piecewise rate function.
- The spatial dynamics among multiple locations are characterized by interactions of multiple inhomogeneous Poisson processes in a network.
- With one reasonable assumption, the dynamic network is approximated with a Markov network so that the parameters describing the temporal and spatial dependence can be estimated in a tractable computational complexity.
- An efficient genetic algorithm is designed to infer the epidemic topology.

We apply our model on the surveillance data from the 2009 H1N1 pandemic in Hong Kong. It is generally very difficult to verify the inferred network topology from real data because the true epidemic network topology is unknown and it may vary for different types of infectious diseases for the same population. In this work, we propose a new method based on model selection. Our intuition is that if the epidemic network plays an important role in the disease spread, then the heterogenous network model will describe the data better than the homogenous model without considering the epidemic network. Furthermore, the more similar is the network topology to the true one, the better does the model fit the data. Both inferred epidemic networks display significant effects on the propagation of infectious diseases. Therefore, our findings may help policy makers reduce the risk of future epidemics. Besides the study of epidemics, the model developed in this project can be extended to study a wide range of propagation patterns in other complex systems such as the Internet and World Wide Web (WWW), where individuals form multiple communities through which information can propagate in a similar way as the infectious disease does. We believe our work can contribute theoretically and empirically to both computing science and epidemiology.

There are some limitations in our proposed network model. First, our model only focuses on the disease spread within the network and does not consider the imported cases. The parameters may be over-estimated if the number of imported cases is large. One possible solution is to create a pseudo node in our stochastic network model, which imports some infected cases to the network from time to time. Second, the network structure in our model is static - the connections remain constant over time. This may be a problem for the long-term disease spread because the behaviour of a sub-population may change markedly as a consequence of an outbreak. The possible solution is to design a new online optimization algorithm that progressively estimates the network topology over time. Third, our SIR-based model is only suitable for the situation where the susceptible population maintains a relatively constant size and structure. To model malaria transmission where asymptomatic infection plays a central role, the SIR-based model is not a good candidate. We will investigate them in our future work.
